# Receptor for Activated C Kinase1B (OsRACK1B) Impairs Fertility in Rice through NADPH-Dependent H_2_O_2_ Signaling Pathway

**DOI:** 10.3390/ijms23158455

**Published:** 2022-07-30

**Authors:** Md Ahasanur Rahman, Herman Fennell, Hemayet Ullah

**Affiliations:** 1 Department of Biology, Howard University, Washington, DC 20059, USA; md.rahman@bison.howard.edu; 2 Department of Biological Sciences, Hampton University, Hampton, VA 23668, USA; herman.fennell@hamptonu.edu

**Keywords:** OsRACK1B, NADPH oxidase, Rboh, ROS, H_2_O_2_ signaling, pollen, fertility, rice

## Abstract

The scaffold protein receptor for Activated C Kinase1 (RACK1) regulates multiple aspects of plants, including seed germination, growth, environmental stress responses, and flowering. Recent studies have revealed that RACK1 is associated with NADPH-dependent reactive oxygen species (ROS) signaling in plants. ROS, as a double-edged sword, can modulate several developmental pathways in plants. Thus, the resulting physiological consequences of perturbing the RACK1 expression-induced ROS balance remain to be explored. Herein, we combined molecular, pharmacological, and ultrastructure analysis approaches to investigate the hypothesized connection using T-DNA-mediated activation-tagged RACK1B overexpressed (OX) transgenic rice plants. In this study, we find that OsRACK1B-OX plants display reduced pollen viability, defective anther dehiscence, and abnormal spikelet morphology, leading to partial spikelet sterility. Microscopic observation of the mature pollen grains from the OX plants revealed abnormalities in the exine and intine structures and decreased starch granules in the pollen, resulting in a reduced number of grains per locule from the OX rice plants as compared to that of the wild-type (WT). Histochemical staining revealed a global increase in hydrogen peroxide (H_2_O_2_) in the leaves and roots of the transgenic lines overexpressing OsRACK1B compared to that of the WT. However, the elevated H_2_O_2_ in tissues from the OX plants can be reversed by pre-treatment with diphenylidonium (DPI), an NADPH oxidase inhibitor, indicating that the source of H_2_O_2_ could be, in part, NADPH oxidase. Expression analysis showed a differential expression of the NADPH/respiratory burst oxidase homolog D (RbohD) and antioxidant enzyme-related genes, suggesting a homeostatic mechanism of H_2_O_2_ production and antioxidant enzyme activity. BiFC analysis demonstrated that OsRACK1B interacts with the N-terminal region of RbohD in vivo. Taken together, these data indicate that elevated OsRACK1B accumulates a threshold level of ROS, in this case H_2_O_2_, which negatively regulates pollen development and fertility. In conclusion, we hypothesized that an optimal expression of RACK1 is critical for fertility in rice plants.

## 1. Introduction

The receptor for Activated C Kinase1 (RACK1) is a WD-40 type transmembrane protein that is ubiquitously expressed in different tissues and organs in plants and participates in several biological processes ranging from seed germination, root formation, growth, leaf vegetation, flowering, and fruit ripening to senescence [1,2,3,4]. As a scaffold protein, RACK1 regulates several signaling pathways through interactions with hundreds of proteins in eukaryotic systems [5]. Although it is less clear how RACK1 contributes to diverse biological processes, a dynamic and inducible modification at both the transcriptional and the translational levels has been proposed as a key to RACK1′s functionality [6,7,8]. Early genetic studies demonstrated RACK1′s role in environmental stress signaling and hormone responses. Current evidence focuses on a new insight into RACK1′s role in plant immune signaling and has identified RACK1 as a regulator of reactive oxygen species (ROS)-mediated systemic signaling. For example, rice RACK1 (OsRACK1) is shown to be involved in the immune response against pathogen attack through enhanced reactive oxygen species (ROS), such as H_2_O_2_ production regulated by the Rac1 immune complex [9]. OsRACK1 forms a complex with the GTP form of Rac1 and triggers NADPH oxidase-dependent ROS bursts (in plants, NADPH oxidase (NOX) is also known as respiratory burst oxidase homolog, Rboh) in response to blast fungus. At the same time, Li et al. showed that RACK1 negatively regulates the redox system-related tolerance to drought stress in transgenic rice plants, and superoxide dismutase activity (SOD) is significantly higher in *rack1a* mutant plants than in normal plants [10]. Zhang et al. have shown that OsRACK1 positively regulates rice seed germination by controlling endogenous hydrogen peroxide (H_2_O_2_), and several Rboh transcripts were differentially expressed in transgenic rice plants [4].

RBOHs encode NADPH oxidases that localize at the plasma membrane to produce O_2_^•–^, which then mostly converts to apoplastic H_2_O_2_ [11,12,13]. Studies have demonstrated that the spatiotemporal expression of RBOH proteins inducing ROS bursts is highly regulated by multiple factors, such as Ca^2+^ influx, phosphorylation by calcium-dependent protein kinases (CPKs) and mitogen-activated protein kinase (MAPK) elements, transcriptional reprogramming, and phytohormone regulation [14,15]. The evidence also suggests that cytosolic calcium concentration plays an important role in modulating the NADPH oxidase activity by binding the EF-hand motifs of the N-terminal region of RBOH [16]. A tight balance between the dynamic regulation of RBOH proteins and the ROS bursts is shown to be essential for plant growth and development, specifically for pollen development, pollen tube elongation, and cell wall integrity [17,18,19,20,21,22,23].

The first *RBOH* gene was characterized in rice (*OsRbohA*) by Groom et al. [24], and as of now, multiple homologues have been identified in plant lineages. For example, the *Arabidopsis thaliana* genome has ten and rice has nine *RBOH* genes, and only four *RBOH* genes have been reported in *Zea mays* (maize) [13,16,25]. All *RBOH* genes play important roles in various biological and stress-related processes in plants, including seed germination, root hair formation, senescence, systemic signaling, pollen tube growth, and pathogen response or environmental stresses, such as salinity, drought, etc. [12,13,18,21,22,26,27,28,29,30,31,32,33]. For instance, RbohD is ubiquitously expressed in all tissues and has been implicated in stress responses, immunity, endosperm development, and cell wall damage-induced lignification [13,27,34,35,36,37,38].

Three separate studies in maize, soybean, and kale suggest that the overexpression of RACK1 induces Rboh-dependent H_2_O_2_ production to regulate pathogen, drought, and salt resistance, respectively [39,40,41]. These reports uncovered shared signaling components and a direct crosstalk between RACK1 and H_2_O_2_ signaling cascades in response to environmental cues. Yet, the dynamics and physiological outcomes of such complex interactions remain elusive. The rice genome contains two RACK1 homologous genes: OsRACK1A and OsRACK1B with 82% amino acid sequence homology [9].

In this study, we used the rice T-DNA insertion activation-tagging mutagenesis approach to characterize the function of OsRACK1B. We explored a possible mechanism of how RACK1 cross-talks with ROS signaling components and its potential physiological outcome. We found that the overexpression of OsRACK1B induces an Rboh-mediated ROS burst as a form of H_2_O_2_ through direct interaction with the N-terminal region of RbohD. Our work indicates that the apoplastic systemic ROS produced by NADPH oxidases, together with the controlled antioxidant enzyme activity, is critical to ensuring the proper timing of anther dehiscence, pollen viability, and pollen cell wall integrity. We hypothesize that an optimal level of OsRACK1B is essential for pollen development and that OsRACK1B helps to maintain pollen cell wall integrity by maintaining a threshold level of H_2_O_2_.

## 2. Results

### 2.1. Identification of T-DNA Insertion Activation-Tagged Rice Plants Overexpressing OsRACK1B

**Figure 1:**
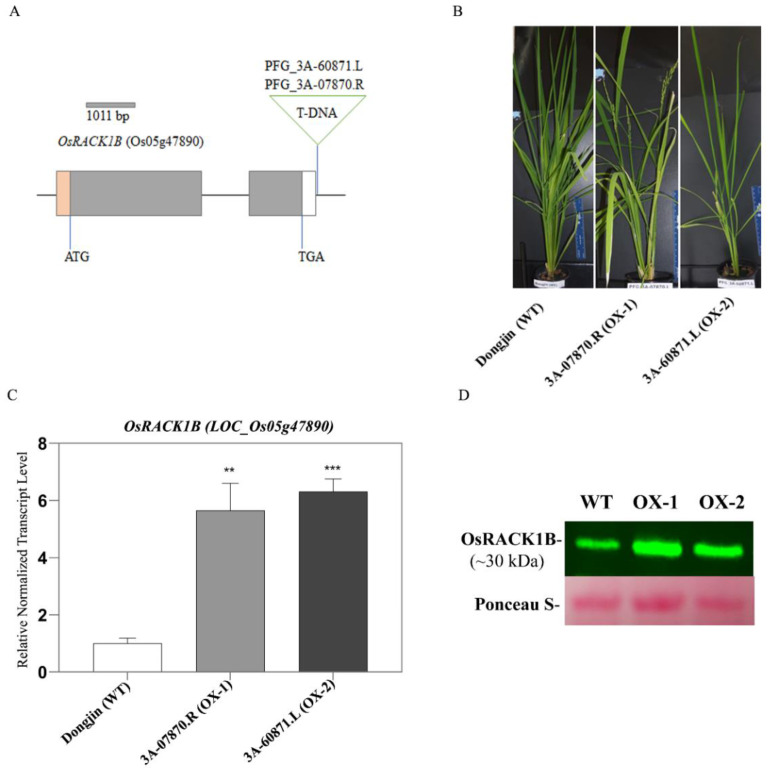
Characterization of T-DNA insertion lines with analysis of gene expression and protein abundance. (**A**) Schematic diagram depicting the positions of T-DNA insertions in OsRACK1B. Gray, orange, and white bars represent the exons, 5’-UTR, and 3´-UTR region, respectively. ATG and TGA are start and stop codons. The 1011 bp indicates the CDS length. The gray line represents the intron. The triangles indicate OsRack1b mutant alleles (OX-1, PFG_3A-07870.R; OX-2, PFG_3A-60871.L). Plants with T-DNA insertion in 3A-60871.L and 3A-07870.R lines (**B**) resulted in RACK1B overexpression, revealed by expression analysis shown in qRT-PCR (**C**) and Western blot (**D**). Samples were collected from four-week-old wild-type (WT) and OsRack1b mutant rice plants. For Western blot, an equal amount of leaf proteins extracted from the indicated genotypes was subjected to immunoblot analysis stained with anti-OsRACK1B antibody. Ponceau-stained membrane is shown as the loading control. Molecular weight markers are indicated in kDa. For qRT-PCR analysis, the normalized expression level of OsRACK1B in transgenic rice lines was compared to the wild-type plant. Total RNA was extracted from leaf tissues sampled from detached leaves of four-week-old wild-type and transgenic plants, as described in Methods. OsActin-1(LOC4333919) was used to standardize transcript levels in each sample. The data are shown as the means  ±  SE of three technical repeats. Double, and triple asterisks indicate statistical significance: ** *p* < 0.01 and *** *p* < 0.001, respectively, compared to wild-type (Student’s *t*-test). Primers are listed in Table S2.

For the functional characterization of OsRACK1B (Loc_Os05g47890), we screened overexpression lines from the RICEGE database (http://signal.salk.edu/cgi-bin/RiceGE, accessed on 4 April 2012). We identified the PFG_3A-60871.L, PFG_3A-07870.R lines as the putative gain-of-function lines where a quadruple CaMV 35S enhancer is located at 3′-UTR of Loc_Os05g47890 on chromosome 5 in opposite directions. PCR genotyping analysis confirmed the presence of T-DNA in several seedlings. Expression analysis by qRT-PCR and Western blot revealed that two plants from putative gain-of-function lines accumulated 5–7-fold more OsRACK1B transcripts and higher protein levels than those in Dongjin (hereafter termed WT) plants (Figure 1B,D). We refer to these two lines as the OsRACK1B overexpressed lines and designated them OX-1 (PFG_3A-07870.R) and OX-2 (PFG_3A-60871.L). Sequence analysis indicated that in both plants the T-DNA insertion was located in the 3′UTR region after the stop codon (TGA) at the 3′end (Figure 1A, schematic diagram). Within the nearly 10 kb insertion site, except for several transposons, no other gene coding sequences could be seen, implying a low possibility of interference by the ectopic activation of other nearby genes.

### 2.2. OsRACK1B Modulates RBOH-Dependent H_2_O_2_ Accumulation

**Figure 2:**
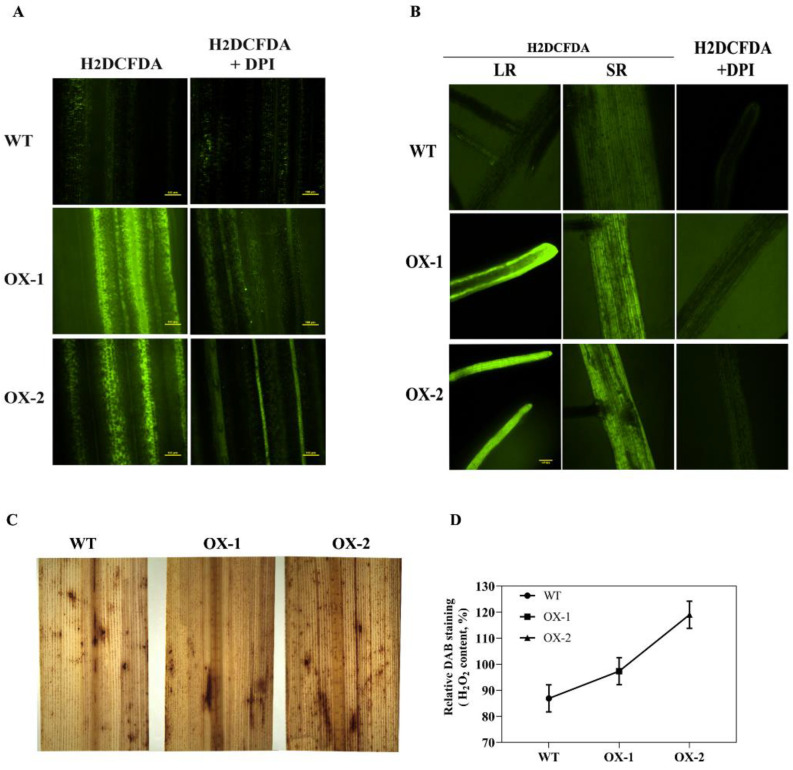
Representative images showing the effect of altered expression of OsRACK1B on H_2_O_2_ accumulation in rice leaves (**A**,**C**) and roots (**B**). (**A**,**B**) H_2_DCFDA staining (green fluorescence) alone or plus DPI of the leaf sheaths (50 µM) and roots (10 µM) of eight-week-old wild-type and OsRACK1B-overexpressed rice plants. A fluorescence microscope was used for the detection of H_2_O_2_ fluorescence. LR, lateral root; SR, seminal root. Bars = 100 μm. (**C**,**D**) DAB staining shows DAB polymerization product (dark brown spots) in the fully expanded leaf of eight-week-old wild-type and OsRACK1B-overexpressed (OX) rice plants (**C**) and the relative quantity of spots developed after 72 h of DAB staining measured by ImageJ (imagej.nih.gov/ij, accessed on 31 October 2019).

Compelling evidence has indicated that the overexpression of RACK1 in rice (*OsRACK1A*), maize (*ZmRACK1*), kale (*BoRACK1*), and soybean (*GmRACK1*) plants leads to elevated ROS production [39,40,41]. Therefore, we investigated the oxidation states of the transgenic rice plants using 3,3′-diaminobenzidine (DAB) and 2′,7′ dichlorodihydrofluorescein diacetate acetyl ester (H_2_DCFDA) staining methods to determine the accumulation of H_2_O_2_ in transgenic and wild-type plants. We found that leaves from OsRACK1B-overexpressed plants accumulate more H_2_O_2_, visible as dark brown spots due to H_2_O_2_-specific DAB polymer precipitation [42], than that of the wild-type plants (Figure 2C). To investigate the source of the intracellular accumulation of H_2_O_2_, we stained leaves with H_2_DCFDA alone and with diphenyleneiodonium (DPI), which specifically inhibits superoxide radical generation by RBOH proteins [27,43]. Consistent with the DAB staining, microscopic analysis of the H_2_DCFDA staining (green fluorescence) revealed significant H_2_O_2_ accumulation inside the cells of RACK1B-OX rice leaf sheaths and roots compared to that of the wild-type (Figure 1A,B). However, OsRACK1B-OX leaf sheaths and roots that were pretreated with DPI showed a distinct inhibition of H_2_O_2_ accumulation. These findings suggested that RACK1B activates the Rboh-dependent H_2_O_2_ production.

### 2.3. OsRACK1B Regulates Rboh and SOD Activity

**Figure 3:**
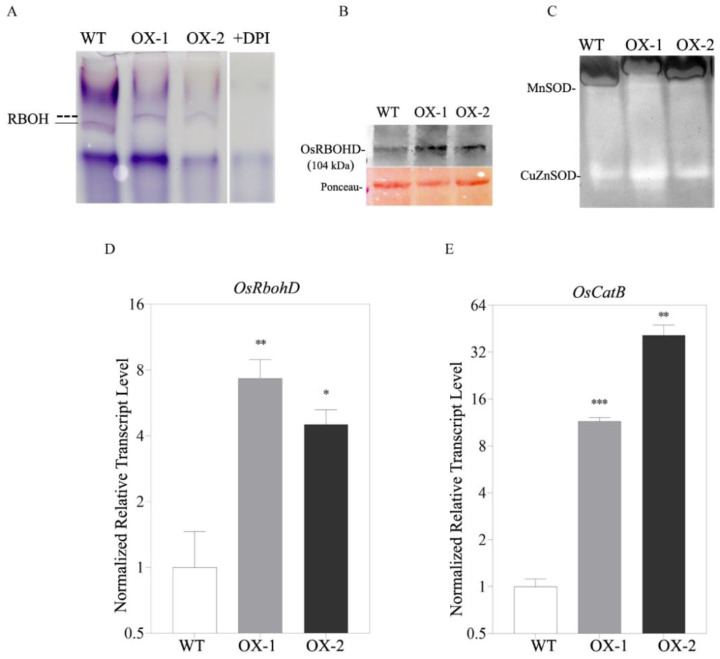
OsRACK1B alters the Rboh and antioxidant activity. (**A**) Native PAGE (in-gel) analysis of Rboh activity. Rice leaves from OsRACK1B overexpressed and WT plants were fractionated by denaturing PAGE and assayed for Rboh activity. Fifty micromolar of Rboh inhibitor, DPI, was added to the microsomal fraction of leaf from wild-type rice plant before loading in the designated well of the native PAGE gel indicated in the Rboh activity gel. The straight and dotted lines in RBOH activity gel indicate the different migration patterns of the Rboh enzyme. (**B**) Western blot analysis of OsRBOHD protein showing higher accumulation of the protein compared to the WT. An equal amount of leaf proteins extracted from the indicated genotypes was subjected to immunoblot analysis stained with anti-OsRBOHD antibody. Ponceau-stained membrane is shown as the loading control. Molecular weight markers are indicated in kDa. (**C**) The activity of SOD isozymes; MnSOD and CuZnSOD from total proteins extracted from OX and WT plants. SODs were separated by native PAGE on 12% (*w*/*v*) polyacrylamide gels, and gels were stained by the photochemical nitroblue tetrazolium method and scanned using flat-bed scanner. (**D**,**E**) qRT-PCR analysis shows elevated transcript levels of *OsRbohD* and *OsCatB* genes. The transcript levels were normalized using *OsActin-1* (LOC4333919). The data are shown as the means  ±  SE of three technical repeats. Single, double, and triple asterisks indicate statistical significance: * *p* < 0.05, ** *p* < 0.01, and *** *p* < 0.001, respectively, compared to wild-type (Student’s *t*-test). Primers are listed in Table S2.

Given that H_2_O_2_ accumulation was observed in OsRACK1B-OX plants, we investigated the in-gel activity assay of crude protein extracts from the leaves of transgenic and WT plants. Interestingly, the gel activity using NAPDH as a substrate revealed a different migration pattern in the OsRACK1B-OX samples than that of the wild-type (Figure 3A). While the WT fractionated samples produced a formazan band of 80 to 75 kD molecular masses, the OsRACK1B-OX samples had approximately 103 kD-sized bands. As the RBOH activity assay is based on the migration of enzyme complexes through native gels, we conclude that the RBOH enzyme activity in OsRACK1B-OX leaves is different to that of the wild-type plant. To further confirm the Rboh assay, the WT sample was tested for sensitivity to DPI (Rboh/NADPH oxidase inhibitor, 50 µM), and we observed a complete inhibition of Rboh activity (Figure 3A, lane 4, +DPI). These data provide further evidence that OsRACK1B is regulating OsRboh activity, possibly at the post-transcriptional or post-translational levels. Remarkably, several reports suggested that overexpression of Rboh does not result in constitutive ROS production but, rather, a post-transcriptional or post-translational processing such as phosphorylation that activates Rboh for the ROS burst [13,37,44,45,46]. We further examined the transcription levels of two previously reported rboh genes: *RbohB* and *RbohD*, which are responsible for ROS production in rice. The qRT-PCR results revealed that the *RbohD* transcript levels were significantly increased in RACK1B-OX plants (Figure 3D), while the *RbohB* expression (Supplemental Figure S3) was lower than that of the WT plant. We further confirmed the increased expression of OsRBOHD protein in both RACK1B-OX plants compared to WT, using Anti-RBOHD antibody (Figure 3B).

Rboh activation is attributed to the reactive O_2_^•–^ that is immediately dismutated by superoxide dismutase (SOD) enzymes to form H_2_O_2_ in the cytoplasm, mitochondria, and chloroplasts [47,48]. Therefore, we examined two important scavenging enzymes: MnSOD and CuZn-SOD activity. Manganese containing SOD (MnSOD) is localized in the mitochondria; copper and zinc-containing SOD (CuZnSOD) is located in the cytoplasm and the nucleus [49,50]. In-gel analysis revealed that the CuZn-SOD and MnSOD activities in the transgenic samples were more elevated than those of the WT plant (Figure 3C). Catalase possesses a high affinity for H_2_O_2_ and catalyzes its dismutation into H_2_O and O_2_. Our qRT-PCR analysis showed that *Catalase B* transcripts were induced in transgenic plants compared to those of the WT plant (Figure 3E). Combinedly, these data corroborate our observation of excessive H_2_O_2_, which is partly due to the increased activity of the antioxidant enzyme activities.

### 2.4. OsRACK1B Interacts with N-terminus RbohD

**Figure 4:**
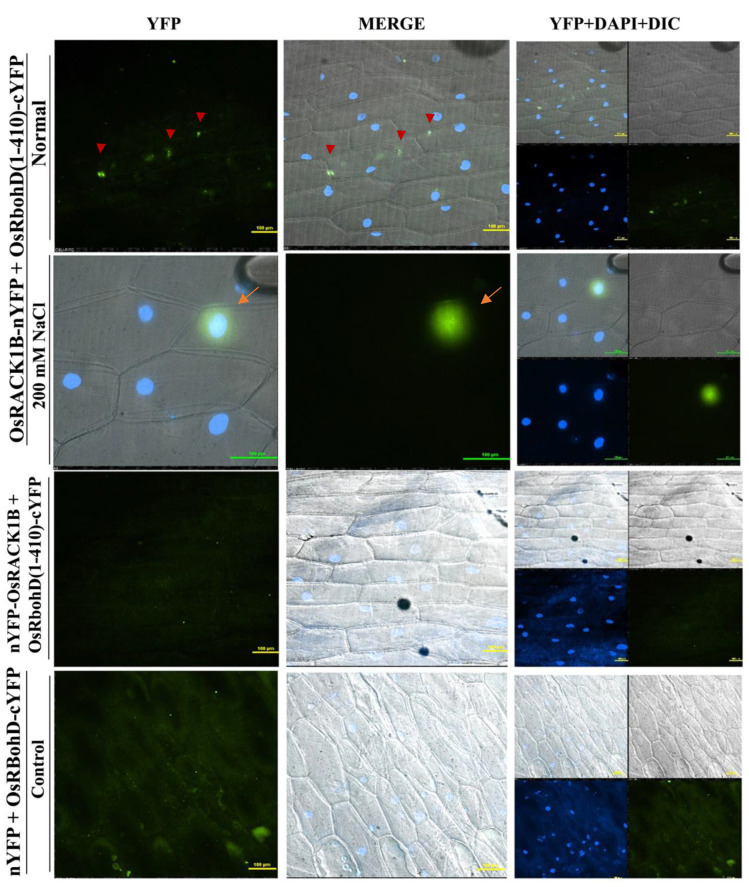
Visualization of OsRACK1B and OsRbohD interactions using bimolecular fluorescence complementation (BiFC) assay. OsRACK1B interacts with the N-terminus of OsRbohD inside the cytoplasm (red arrowhead) under normal conditions (top panel) and inside the nucleus (orange arrow) in the presence of salinity stress (second panel). In the control, the coexpression of OsRACK1B and OsRbohD (1-410 aa) constructs showed DAPI fluorescence but no YFP signals (bottom two panels). The onion epidermal cells were transiently transformed with the full-length OsRACK1B cds and N-terminal region of OsRbohD consisting of 1 to 410 amino acid sequences. Blue fluorescence from DAPI (4 9, 6-diamidino-2-phenylindole) staining indicates nuclei, merge, merged images of YFP channel, DAPI, and differential interference contrast image (DIC). Constructs were fused to either carboxy (YFP-C) or amino (YFP-N) terminus of YFP and vice versa. All constructs were under the control of the 35S promoter. Each pair of recombinant plasmids encoding nYFP and cYFP fusions was mixed 1:1 (*w*/*w*) and co-bombarded into onion epidermal cell layers. The transformed onion epidermal layers were incubated at 22 °C for 16–24 h. For plasmolysis, epidermal cells were incubated in liquid MS media containing 200 mM NaCl for 30 min following bombardment to separate cytoplasm from cell walls. The coexpression of nYFP with OsRbohD (1-410 aa)-cYFP serves as a negative control as there are no YFP signals observed. Bars = 100 μm.

Next, we investigated whether OsRboh is regulated by OsRACK1B through direct interaction in plants using bimolecular fluorescence complementation (BiFC) assays. Crystal structure and mutational analysis revealed that Rboh protein activity is regulated through its conserved cytoplasmic N-terminal region containing two EF-hand motifs [51]. In vitro binding and NMR assay revealed that the N-terminal region of RBOH forms intramolecular interactions in the C-terminal region to prevent homodimerization [51,52,53]. Indeed, using split ubiquitin two-hybrid assays, Nakashima et al. [9] have shown that full-length OsRbohB did not interact with OsRACK1A, and the author hypothesized that the C-terminus of RbohB has an inhibitory effect on the interaction. The reports indicated that among all isoforms, respiratory burst oxidase homologue D (RbohD) is the most active and highly expressed for apoplastic ROS generation [52,54]. We also found elevated RbohD transcription in OsRACK1B-OX plants (Figure 3D). Therefore, to further verify our observation, we deliberately chose the N-terminal region of OsRbohD encompassing amino acid 1-410, which was cloned with the C-terminal part of YFP, and the full-length OsRACK1B was fused with both the N- and the C-terminal part of YFP for our BiFC experiment. We observed fluorescence in the cytoplasm indicative of protein–protein interactions in normal non-stressed onion cells bombarded with OsRACK1B-nYFP and OsRbohD (1-410)-cYFP constructs in the BiFC assays (Figure 4, top panel and Supplemental Figure S4A). To examine whether the expression of both OsRACK1B and OsRbohD can be induced by environmental stimuli such as NaCl treatment, the epidermal cells were incubated with Murashige and Skoog medium containing 200 mM NaCl for 30 min. Indeed, a strong YFP fluorescence was detected in the nucleus of the plasmolyzed cells (Figure 4, second row and Supplemental Figure S4B), suggesting that OsRACK1B interacts with the OsRbohD in the cytoplasm in a physiological condition, but the complex moves into the nucleus in response to environmental stimuli. The trigger for such shuttling between the cytoplasm and nucleus, however, remains to be determined.

### 2.5. Overexpression of OsRACK1B Affects Fertility, Anther Dehiscence, and Spikelet Fertility

**Figure 5:**
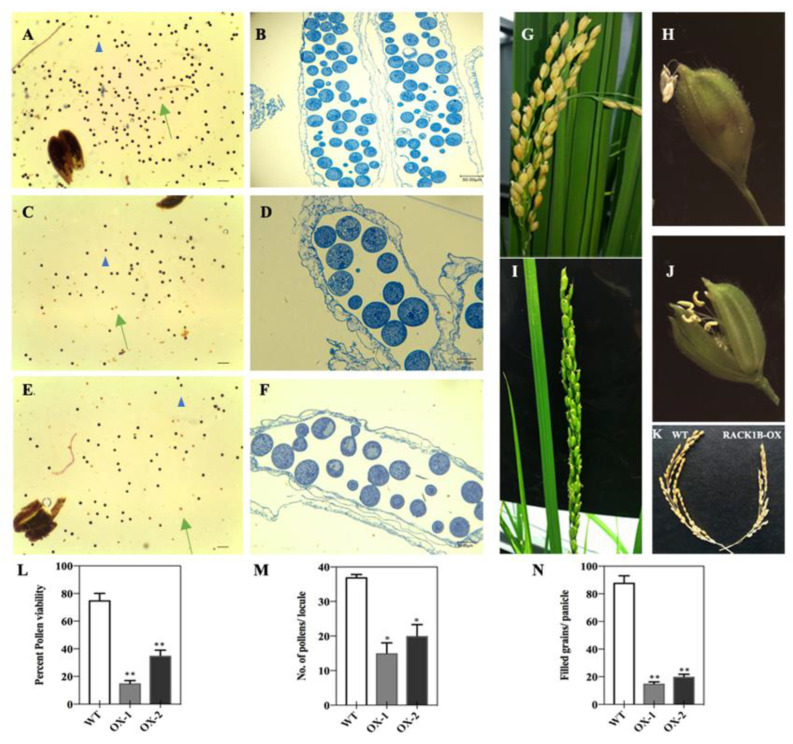
Analysis of the pollen phenotype from wild-type (WT) and OsRACK1B-OX transgenic plants. (**A**,**C**,**E**), KI-I_2_ staining showing reduced pollen viability in the OX-1 (**C**) and OX-2 (**E**) plants compared to that of the WT plant (**A**). Blue arrowheads indicate normal pollen (deep purple color grains), while green arrows indicate abnormal pollen (weak red color grains). Percentage pollen viability (**L**) was calculated based on the different colors of pollens from (**A**,**C**,**E**). Bars = 100 μm. (**B**,**D**,**F**), transverse section analysis of single locule form anther of WT (**B**), OX-1 (**D**), and OX-2 (**F**). Anthers were stained with toluidine blue at mature pollen stage. Number of pollen grains per locule was calculated (**M**). Bars = 50 μm. (**G**,**I**,**K**) Panicles of WT (**G**) and OsRACK1B-OX (**I**) plants. M, quantitative analysis of grains per locule of anthers from WT and RACK1B-OX plants. (**H**,**J**) Spikelet phenotype of WT (**H**) and OsRACK1B-OX (**J**) plants. (**K**) Panicle phenotype of WT and RACK1B-OX plants. No. of filled grains per panicle was calculated (**N**). Data are means ± SEM of three biological replicates. Singleand double asterisks indicate statistical significance: * *p* < 0.05, ** *p* < 0.01, respectively, compared to wild-type (Student’s *t*-test).

It is known that RACK1 is involved in multiple developmental processes, including plant growth, leaf production, and flowering [1,4]. In our study, one of the most pronounced features of the OsRACK1B-OX line was that the plants displayed reduced fertility with shorter floral organs, including panicles, spikelets, palea, lemma, and anthers, than those of the wild-type plants. To further characterize the role of RACK1B in rice, we then investigated the fertility rate and pollen development of the OsRACK1B-OX transgenic rice plants. We examined the flowering and fertility phenotypes of OsRACK1B-OX and WT plants. The microscopic observation of the female reproductive organs appeared normal. Therefore, to investigate whether the reduced fertility was due to pollen viability, we used KI-I_2_ staining which reflects the amount of starch accumulated in the pollen and is highly correlated with pollen fertility [55,56]. Starch provides energy for pollen maturation and pollen tube germination [57]. In general, viable pollens are stained a dark purple color due to enough starch presence, whereas those lightly stained or unstained are considered non-viable. We found that approximately 20–40% of the pollens from the OsRACK1B-OX plants were darkly stained, compared with >80% of the pollens from the wild-type (Figure 5A,C,E,L). As shown in Figure 5G,I,N, the seed-setting rate of the OsRACK1B-OX plants was only 20–30%, nearly a quarter that of the wild-type (85%), which is consistent with abnormal pollen morphology. To gain further insights, we performed a detailed morphological analysis of the anthers at the flowering stage of the transgenic rice plants. A semi-thin transverse section using toluidine blue staining revealed that OsRACK1B-OX plants had a shrinking anther and a smaller pollen sac than that of the wild-type anthers (Figure 5B,D,F). In addition, the number of pollen grains per locule was only 10–20 grains per locule in RACK1B-OX anthers, less than half that of the wild-type (40 grains per locule) (Figure 5M). We also observed abnormality in anther dehiscence and flower opening in the OsRACK1B-OX plants. In the wild-type, the anthers dehisced completely and shed pollen before the spikelet began to close (Figure 5H). After complete dehiscence of the anthers, the spikelet closes, keeping the empty anthers outside the spikelet. However, we observed that most of the spikelets of the OsRACK1B-OX plants remained open during anthesis, and the anthers remained indehiscent (Figure 5J). This observation suggested that the reduced spikelet fertility is due, at least in part, to the inability of the anthers to dehisce, the low viability of the pollen grains, and defects in spikelet closing. Combinedly, our analysis implied that OsRACK1B plays a critical role in panicle development and anther dehiscence in rice.

### 2.6. OsRACK1B-OX Plants Exhibit Defective Pollen Morphology and Delayed Dehiscence

Generally, anther dehiscing starts when the anthers reach the top of the spikelet and release pollen grain over the stigma right before the spikelet opening. After anthesis, the filaments elongate further. As soon as the remaining pollen grains are released, the spikelet closes, keeping the empty anthers outside the spikelet (Figure 5H). Unlike the wild-type plant, the anthers of the RACK1B-OX plants did not dehisce at the time of spikelet opening (Figure 5J). While other the reproductive organs appeared normal, we assumed that the spikelet sterility of the RACK1B-OX plants was due mainly to the inability of the anthers to dehisce and release pollen grains normally.

**Figure 6:**
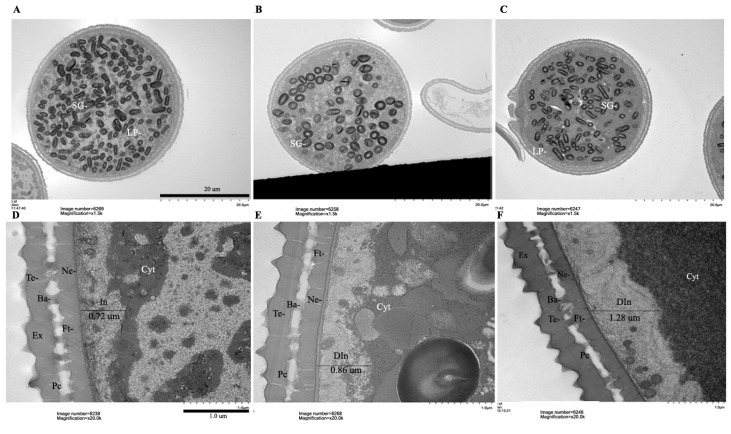
Transmission electron microscopy (TEM) examination of wild-type (WT) and OsRACK1B-OX transgenic pollen grains during anther dehiscence stage. (**A**–**C**) TEM image showing that the WT pollen grain (**A**) contains a larger number of starch granules than OX-1 (**B**) and OX-2 (**C**) pollen. (**D**–**F**) Higher magnification of the pollen wall showing normal pollen wall from the WT (**D**) and the abnormality of the pollen wall in OX-1 and OX-2 mutants (**E**,**F**). (**D**) A higher magnification TEM image of the normal pollen wall from (**A**). (**E**) A higher magnification image of the defective pollen wall from (**B).** (**F**) A higher magnification image of the pollen wall from (**C**). SG, starch granules; LP, lipid bodies; Ba, bacula; Ne, nexine; Cyt, cytoplasm; Ex, exine; In, intine; Din, defective intine; Pc, pollen coat; Ft, foot layer; Te, tectum; bars = 20 µm in (**A**–**C**), 1 µm in (**D**–**F**).

To better understand the structure of the defective pollens during pollen development, we used transmission electron microscopy (TEM) analysis for the structure of the pollen grains from both the wild-type and the OsRACK1B-OX plants. At the mature stage, the most obvious difference between pollen sources was in the number of starch granules inside the pollens. Whereas the wild-type pollen grain had accumulated abundant starch granules (Figure 6A), the OsRACK1B-OX pollen grains had decreased starch granules with irregular shapes (Figure 6B,C). This observation was corroborated with our KI-I_2_ staining experiment. Other apparent abnormalities were in the vacuole size, being smaller in the wild-type (Figure 6A,D), but remaining much larger in the OX lines (Figure 6B,C,E,F). In addition, abnormality of the cytoplasm density was apparent in the OsRACK1B-OX pollens (Figure 6E,F) compared to that of the wild-type (Figure 6D). Interestingly, DAPI staining of aborted pollens from the anthers that showed indehiscence or delayed dehiscence revealed that almost all the pollen grains (from OsRACK1B-OX) contained three, two, one, or no DAPI-stained nuclei in intact or completely collapsed forms with callose patches on the pollen wall (Supplemental Figure S5A,B).

The pollen wall consists of several layers: exine in the outer layer, which includes tectum; bacula links exine with the inner layer intine, which includes nexine, the pollen coat, and the foot layer [58,59,60,61]. The intine development is similar to that of plant cells and is made of celluloses, pectin, hydrolytic enzymes, and hydrophobic proteins, required to maintain the structural integrity of the pollen grains, pollen maturation, and pollen tube growth for fertility [60,62,63,64]. Higher magnification TEM images revealed defective intine development in the OsRACK1B-OX pollens compared with that in the wild-type. More specifically, the OX grains accumulated a thicker intine layer (0.86 µm in OX-1 and 1.28 µm in OX-2) compared with that of the wild-type pollen grain (0.72 µm) (Figure 6D–F). It is hypothesized that abnormalities in intine formation lead to the arrest of pollen development at the maturation stage, resulting in a lack of starch granule accumulation [62,65,66]. These differences are also in agreement with our KI-I_2_ staining and thin-section analysis of wild-type and mutant pollen.

## 3. Discussion

As a scaffold protein, RACK1A has been widely studied and has been implicated in plant growth, development, and stress response pathways, but the functional characterization of RACK1B remains to be explored. In addition, RACK1′s functional role in cereal crops such as rice is still in its infancy. Over the past decades, an increasing number of studies have elucidated RACK1′s contribution to seed germination and defense response mechanisms via ROS signaling cascades [9,10,39,67,68]. Nevertheless, the impact of the overlapping RACK1 and ROS signaling pathways in the context of rice pollen development and fertility have not been examined yet. In this study, we carried out a complex characterization of OsRACK1B through biochemical, histochemical, and ultrastructure analysis.

Emerging evidence suggests that a certain threshold level of ROS, such as H_2_O_2_, can initiate intracellular signaling cascades associated with responses to abiotic and biotic stressors in living organisms [28,31,32,69,70,71,72,73,74,75]. ROS generated inside the cell converts H_2_O_2_ by the dismutation of O_2_, either spontaneously or via superoxide dismutase (SOD) catalysis [76,77]. The mobility, selectivity, and relative stability of H_2_O_2_ make it an ideal molecule to act as a second messenger that transduces retrograde signals from chloroplasts to the nucleus in response to stress [78,79]. While chloroplast, mitochondria, and peroxisomes are considered the major source of metabolic ROS, the reports suggest apoplasts as a site for signaling ROS generation that can cross-talk with other sources in response to stress adaptation [27,80]. The reports indicate that OsRACK1 directly interacts with the N-terminus of the NADPH oxidase (Rboh) and stimulates ROS generation in rice [9]. In agreement with previous findings, we also showed that OsRACK1B interacts with the N-terminal end of RBOH. AtRACK1 was also found to bind CuZn-SOD in split-ubiquitin-based yeast-two-hybrid assays [81]. Considering the intracellular mobility of H_2_O_2_ [82,83], it is tempting to speculate that ectopic expression of RACK1B-activated PM-bound Rboh generates H_2_O_2_ via the action of apoplastic CuZn-SODs, resulting in further biosynthesis of ROS as a systemic wave and generating a positive feedback loop for homeostasis. However, the exact mechanism needs further investigation.

Accumulating evidence suggests that, in contrast to intracellular sources, membrane-localized or apoplastic ROS (in particular H_2_O_2_) can act as a signaling molecule at a threshold level to regulate growth and development and adaptation to various biotic and abiotic stresses [73,84,85,86,87,88]. H_2_O_2_ is recognized as the central molecule in redox signaling due to its long lifespan, low reactivity, membrane permeability, mobility from its generation site to a target site, and stability compared to singlet O_2_ [71,89]. Under normal physiological conditions, H_2_O_2_ production is tightly controlled and fine-tuned by ROS scavenging enzymes and low molecular weight components. RBOH/NADPH oxidase is known as the major source of ROS, presumably H_2_O_2_, in plant cells. Structural analysis suggests that RBOH consists of six transmembrane domains, NADPH hydrophilic domains, two N-terminal Ca^2+^-binding EF-hand motifs that include a GTPase binding region, a C-terminal dehydrogenase domain that binds flavin adenine dinucleotide (FAD), and a functional oxidase domain responsible for superoxide production by transferring electrons from NADPH to oxygen [51,90]. Studies in different plant species have shown that post-transcriptional or post-translational regulation is crucial for Rboh activity [15,37,46,52]. The N-terminal region undergoes post-translational modification by several protein kinases, such as calcium-dependent protein kinases (CDPKs) and calcineurin B-like protein-interacting protein kinases (CIPKs), or Ca^+^-independent kinases, such as receptor-like cytoplasmic kinases (RLCKs), plasma-membrane-associated kinase BIK1 (Botrytis-induced kinase1), and mitogen-activated protein kinases (MAPKs) [37,46,91]. Interestingly, Rboh activity can also be regulated by its interacting partners, such as OsRACK1 [9] and 14-3-3 proteins [92]. Our experiment suggests that OsRACK1B binds to the N-terminus cytoplasmic region of the RbohD. Therefore, we speculate that this binding makes it susceptible to post-translational modification, and OsRboh in OsRACK1B-OX lines has more opportunity to bind and allow post-translational modification by other proteins as well as allow the ROS generation. We also hypothesize that the RBOH enzyme is undergoing a post-translational modification and may contain different or additional phosphorylation or sumolyation subunits that are responsible for this shift in its enzymatic activity [30,37,93,94].

In our BiFC analysis, we observed that OsRACK1B and OsRbohD interact in the cytoplasm in onion epidermal cells, which is expected given that both proteins were reported to be localized in the cytoplasm in plants [4,9,95]. Intriguingly, the complex translocates to the nucleus in response to salt stress, possibly as a part of a signal transduction pathway to regulate oxidative stress-related gene expression. Transcriptional reprogramming is a hallmark strategy of a defense response in the living system. Oxidative stress such as high salinity provokes large-scale changes in the transcriptomic landscape and generates a systemic ROS wave [27,32,36,96,97]. Plants integrate multiple signals from different cellular compartments to adapt to stress conditions. Retrograde signaling from plastid, mitochondria, cytoplasm, or chloroplast to the nucleus to express a specific set of genes is a common intracellular stress signaling system in a fluctuating environment [28,74,98]. Apoplastic ROS, such as H_2_O_2_ itself as a signaling molecule, instigates a change in gene expression in the nucleus. For instance, the nonexpressor of pathogenesis-related genes 1 (NPR1) protein, a redox-sensitive transcription coactivator, primarily localized in the cytoplasm in both Arabidopsis and tobacco, transported to the nucleus in response to salt stress to function as a coactivator of gene transcription [99,100,101]. Recently, it was shown that a nuclear translocation of GmMPK6 from the cell membrane during high salinity treatment in soybean seedlings can be blocked by DPI treatment, suggesting that the nuclear translocation is mediated by Rboh-dependent H_2_O_2_ [102]. In this aspect, it is unclear whether the OsRACK1B-OsRbohD complex itself moves to the nucleus for gene regulation or H_2_O_2_ produced in response to salt treatment triggers the translocation. Further research will provide more insights on which mechanism the nuclear translocation is mediated in response to oxidative stress.

Most of the studies on Rboh-dependent ROS production have focused on its contribution to the biotic and abiotic stress tolerance in plants. RBOH has also gained attention for its role in seed development, germination, flowering, and other reproductive organs, such as pollens and stamens [20,21,103,104,105].

ROS, in particular H_2_O_2_, has shown to be involved in pollen grain activation in kiwifruit [106,107], hyperpolarization of lily, and tobacco pollen grain protoplast [108,109,110]. Several reports suggest that a tightly controlled spatio-temporal regulation of ROS homeostasis is crucial for pollen development. The reports indicated that excessive accumulation of H_2_O_2_ is responsible for male sterility in rice [23,60,111,112]. During the maturation stages of pollen development, a process called program cell death (PCD) of the tapetum (the highly specialized innermost layer of anther) is essential for pollen development [113,114]. The reports showed that PCD of the Arabidopsis and rice anther tapetum is induced by ROS and leads to the loosening of the pollen grains from surrounding tissues and the development of pollen materials and coatings [61,111]. ROS contributes to the PCD and tapetum degeneration process of pollen development in a time-dependent manner [114]. Therefore, the abnormalities we observed in the pollens in the OsRACK1B-OX plants in our study may be associated with the constitutive accumulation of OsRACK1B-induced H_2_O_2_, leading to the premature initiation of PCD in pollen grains and pollen abortion. We also hypothesize that the defective intine structure is linked to the over-accumulation of H_2_O_2_ as similar findings were observed by Smirnova et al. [106,115]. The author demonstrated that H_2_O_2_ regulates tobacco pollen germination by modifying the pollen wall properties. They also suggest that H_2_O_2_ is responsible for the thickening of the cross-linking structure of the intine layer. It would be interesting to dissect the detailed regulatory mechanisms of how RACK1B contributes to the anther development process.

Pollen wall thickness directly influences pollen longevity [59,116]. The rice pollen wall is generally thin and short-lived compared to other grass species. Rice produces recalcitrant pollen, which remains viable for only a few minutes after anthesis [56,117]. The thin pollen wall in rice facilitates both fast germination on the stigma and dehydration in the air, leading to short longevity [117]. Studies related to pollen wall formation confirmed that pollen wall formation is tightly linked with anthesis, panicle development, and spikelet fertility [118,119,120]. The reduced fertility of the RACK1B-OX plants observed in our study can be explained by the defective architecture of the pollen wall. Indeed, mutation studies on genes important for another development have demonstrated that lack of starch granule accumulation and abnormal intine thickness resulted in male sterility in rice [120,121,122,123]. Our ultrastructure observation suggests that OsRACK1B may also play a direct or indirect role in pollen structure development. However, further studies are necessary to explore the detailed mechanism.

## 4. Materials and Methods

### 4.1. Plant Materials and Growth Condition

We identified OsRACK1B T-DNA insertion lines from the Rice functional genomic express database (http://signal.salk.edu/cgi-bin/RiceGE, accessed on 4 April 2012). T-DNA tagged lines PFG_3A-60871.L and PFG_3A-07870.R. Dongjin (Oryza sativa ssp. Japonica cv. Dongjin) background seeds were purchased from Crop Biotech Inst., Korea [124,125,126].

Seeds were surface sterilized and germinated on full-strength Murashige and Skoog (MS) [127] culture media (Caisson Laboratories, Inc.) at room temperature. Leaves from two-week-old germinated seedlings were used for DNA extraction and genotyping. Wild-type (WT) and selected transgenic lines were then transferred to a hydroponic nutrient solution prepared following the protocol as described by Lakshmanan et al. [128]. Plants were grown at 28 °C during a 14 h light (300 μmol m^−2^ s^−1^) period and at 24 °C during 10 h of darkness with 60% relative humidity. Leaf tissues from the plants were collected, flash-frozen in liquid nitrogen, and stored at −80 °C for protein and RNA extraction.

### 4.2. Genotyping of the T-DNA Flanking Region of OsRACK1B Transgenic Lines

Transgenic lines were screened for possible T-DNA insertions positioned near RACK1B (Loc_Os05g47890) by PCR. T-DNA insertion in the transgenic plants was validated using primers from the left and right border of the T-DNA insert (Supplemental Figure S1), as described by Jeong et al. [125,126]. The LP and RP primers were also used for detecting the *OsRACK1B* gene.

The insertion site of the T-DNA in the transgenic lines was mapped by Thermal Asymmetric Interlaced (TAIL) PCR of the genomic DNA according to previously described protocols (Supplemental Figure S2 and Methods S1) [129]. The PCR products were Sanger sequenced and analyzed using the NCBI BLAST service. All of the primers used for genotyping and TAIL-PCR procedures are listed in Tables S1 and S3.

### 4.3. RNA Extraction, Complementary DNA (cDNA) Synthesis, and Quantitative Real-Time PCR (qRT-PCR) Analysis

Total RNA was extracted from a pool of eight-week-old rice leaves from each plant and reverse transcribed to make cDNA using the SuperScript IV VILO Master Mix kit (Thermo Fisher Scientific, Waltham, MA, USA) following the manufacturer’s instructions.

Quantitative real-time PCR (qRT-PCR) was performed using cDNA and the PowerUP SYBR Green master mix (Thermo Fisher Scientific) with a CFX96 real-time PCR detection system (Bio-Rad, Hercules, CA, USA). Normalized expression (2(-Delta Delta C(T) method) was calculated using the Bio-Rad CFX manager software, employing the housekeeping gene *OsActin1* (Os03g50885) as a reference gene. All experiments were performed in triplicate for technical repeats. The results were plotted as relative values ±SEM and graphically displayed using GraphPad Prism version 8.2.0 (GraphPad Software, Inc., San Diego, CA, USA). The qRT-PCR for each gene was repeated three times. The primer sequences used are listed in Table S2.

### 4.4. Protein Extraction and Western Blot Analysis

Total protein from the leaves was extracted from 100 mg of finely powdered tissue using lysis buffer (CelLytic P, Sigma-Aldrich, St. Louis, MO, USA) containing a protease and phosphatase inhibitor cocktail (Sigma-Aldrich). Total protein content for each sample was quantified by Bradford assay using the Quick Start Bradford dye reagent (Bio-Rad).

Western blot analysis was performed using an equal amount of total proteins per sample and resolved in 4–12% pre-cast XT-MES gel (Bio-Rad). The proteins were transferred to polyvinylidene difluoride (PVDF) membrane and incubated with primary and secondary antibodies. Membranes were developed using the ECL detection kit (Bio-Rad). The signals of the bands were visualized and captured using the Chemi-DocXRS system (Bio-Rad). The OsRACK1B peptide-specific mAb antibodies were raised using two peptides, AGVLRGHNDM and QDLKPEVQAF, corresponding to amino acids 10–19 at the N-terminus and 286–295 at the C-terminus end of the OsRACK1B protein sequences, respectively (Abmart, Shanghai, China). The anti-RBOHD (cat# AS15 2962, sample for validation from Agrisera, Sweden) antibody used in this study demonstrated reactivity as stated on the manufacturer’s website. Ponceau-S (40% methanol (*v*/*v*), 15% acetic acid (*v*/*v*), and 0.25% Ponceau-S) staining of Rubisco was used as the loading control.

### 4.5. Histochemical Staining of Reactive Oxygen Species (ROS)

Diaminobenzidine (DAB) and 2′,7′-dichlorodihydrofluorescein diacetate (H_2_DCFDA) stains were used to detect the in situ H_2_O_2_ level in leaves and roots. The stained tissues were evaluated using compound and spinning disk confocal system microscopes.

The DAB assay was conducted according to the protocol described by Daudi and O’Brien [130]. Briefly, leaves (fully expanded) from eight-week-old wild-type and transgenic plants were excised and floated in the water for an hour to minimize wound-induced ROS production. The samples were vacuum infiltrated in 1 mg/mL DAB-HCL (Sigma-Aldrich, St. Louis, MO, USA) solution for 5 min, followed by incubation in DAB solution for 48 h in darkness at room temperature. Following incubation, the reaction was stopped by washing the leaves with a clearing solution (3:1, 95% ethanol: glacial acetic acid). Chlorophyll from the leaves was removed by boiling with a clearing solution in a six-well plate placed in a water bath (95 °C) for 20 min and photographed using a Leica EZ4 stereo-microscope.

Eight-week-old wild-type and transgenic plant leaves and roots were used for an H_2_DCFDA experiment, as described by Achard et al. [131]. Briefly, the samples were incubated for 30 min at 4 °C in 10 mM H_2_DCFDA (Sigma-Aldrich, St. Louis, MO, USA) (dissolved in MES/KCl buffer), washed with 10 mM MES, 0.1 mM KCl, and 0.1 mM CaCl_2_ (pH 6.0) and left for 60 min at 22 °C. For the DPI treatment, the samples were first treated with DPI (50 µM for leaves and 10 µM for roots, dissolved in DMSO) for 30 min at 22 °C and then incubated with 10 mM H_2_DCFDA for 30 min at 4 °C and washed with the previously mentioned MES buffer. The stained leaves and roots were observed using a Nikon Ti-E-PFS inverted spinning-disk confocal microscope with solid-state laser excitation at 488 nm and emission at 525 nm.

### 4.6. In-Gel Activity Assay of Rboh/NADPH and SOD Enzymes

Native polyacrylamide gel electrophoresis (N-PAGE) was carried out using 4–15% Mini-PROTEAN TGX Stain-Free Gels and native sample buffer (both from Bio-Rad, Hercules, CA, USA) in the Laemmli (1970) buffer system without sodium dodecyl sulfate (SDS). The gels were first run at 20 mA for one hour, after which the electrophoresis buffer was replaced. The gels were then loaded with an equal amount of protein sample and run at 40 mA for 2–3 h to the run-off of the dye front.

Rboh/NADPH native gel assay was performed according to the protocol described by Sagi and Fluhr [104]. For this assay, a plasma membrane (PM) fraction of the leaf extracts was prepared. Freshly harvested leaves were homogenized using homogenization buffer (50 mM HEPES pH 7.5, 0.4 M sucrose, 0.1 M KCl, 0.1 M MgCl_2_, and plant protease inhibitor cocktail (#P9599; Sigma) in the ratio of 1:3 (*w*/*v*) and immediately preserved at 4 °C. The homogenate was centrifuged at 10,000× *g* for 10 min. The supernatant was filtered through Miracloth (Millipore). The microsomal membranes (MF fraction) were separated from the cytosolic fraction (CF) by centrifugation at 30,000× *g* for 60 min at 4 °C. Pellets (MF) were resuspended in 5 mM phosphate buffer, pH 7.8. The NADPH-dependent superoxide anion radical (O_2_^•–^ ) producing capabilities of the membrane fractions were assayed in gels by a modified nitroblue tetrazolium (NBT) reduction method (). Briefly, 20 µg MF protein samples were electrophoresed on a native polyacrylamide gel (4–15% Mini-PROTEAN TGX, Bio-Rad, Hercules, CA, USA), and the gel was incubated in the dark for 20 min in a reaction mixture solution containing 50 mM Tris–HCl buffer (pH 7.5), 0.4 mM NBT (Sigma-Aldrich), 0.1 mM MgCl_2_, and 2.5 mM CaCl_2_. After incubation, 134 µM NADPH was added, and the appearance of blue formazan bands was monitored. The reaction was stopped by immersion of the gels in distilled water and photographed immediately using a flatbed scanner. For control, 50 µM DPI was added to the specific well.

MnSOD and CuZn-SOD native gel activity assay was performed according to the protocol described by Weydert and Cullen [132], with slight modifications. Briefly, the gel was incubated in 2.43 mM NBT for 20 min, followed by soaking in 50 mM phosphate buffer of pH 7.8, containing 28 mM TEMED (Sigma-Aldrich) and 2.8 mM riboflavin for 15 min. The SOD isozymes were visualized as colorless bands as the gel turned blue following exposure to white cool fluorescent illumination until the gel turned blue/purple, and clear achromatic bands appeared. The SOD isozymes were identified using inhibitors, 8 mM H_2_O_2_, and 2 mM KCN. The gels were scanned, and negative images were obtained.

### 4.7. Bimolecular Fluorescence Complementation (BiFC) Assay

For the BiFC assay, full-length OsRACK1B and 1300 bp of RBOHD coding sequences of the N-terminal region were recombined in Gateway-compatible BiFC vectors [133]. Each pair of recombinant plasmids encoding nEYFP or cEYFP fusion proteins was co-bombarded into onion epidermal cells using the Helios DNA particle delivery system (Biolistic PDS-1000/He, BioRad), as described by Hollender and Liu [134]. The onion cells were incubated in MS liquid media for 16–24 h at 22 °C under dark incubation and observed for YFP fluorescence with an inverted spinning-disk confocal microscope (Eclipse Ti-E-PFS, Nikon, Melville, NY, USA). For salt treatment, the onion cells were incubated in liquid MS media with 200 mM NaCl for 30 min before imaging. Confocal fluorescent and DIC images were acquired and processed using the Nikon NIS-Elements software. Negative controls for interaction were provided by empty YFPC and YFPN vectors in combination with the OsRACK1B and RBOHD pSAT expression plasmids (Methods S2) (133). The primers used for BiFC plasmids are listed in Table S4.

### 4.8. Phenotypic Characterization, Pollen Viability Assay, and DAPI Staining

To analyze pollen viability, anthers from mature flowers were dissected before anthesis and fixed in 70% ethanol, and the pollen grains were stained with I_2_–KI staining (0.2% iodine and 2% potassium iodide), as described by Zhou et al. [135]. The stained pollen grains were then photographed with a Leica EZ4 HD microscope. The total number of pollen grains and viable pollen were counted with the software ImageJ (http://rsbweb.nih.gov/ij/, accessed on 12 May 2019). The pollen grains densely stained by the I_2_–KI solution were counted as mature viable pollen.

For 4′,6-diamidino-2-phenylindole (DAPI), single anthers were dissected from the isolated spikelet using a dissecting microscope (Leica EZ4 HD). Mature anthers from the WT and transgenic plants were disrupted on microscope slides using dissecting needles and gently squashed in DAPI staining solution (VECTASHIELD Antifade Mounting Medium with DAPI, Newark, CA, USA) under a cover slip. After staining for 20 min, the fluorescent images were acquired using a Nikon Ti-E-PFS inverted spinning-disk confocal microscope. Photos of the panicles were taken using a point-and-shoot digital camera. Fresh rice spikelets, flowers, and dehiscent anthers were photographed with a Leica EZ4 HD stereo microscope.

### 4.9. TEM and Cross-Section Analysis of Pollen

For transmission electron microscopic (TEM) and cross-section analysis, anthers at the maturation stage from wild-type and RACK1B-OX plants were fixed by immersion in 2% paraformaldehyde-glutaraldehyde fixative in 0.1 M sodium cacodylate buffer (pH 7.2) for 1 h at room temperature. Specimens were rinsed in the same buffer and post-fixed in 2% osmium tetroxide for 3 h, dehydrated in a graded series of ethanol to 100%, and embedded in epoxy resin. For SEM, the anther specimens were thin-sectioned (2–3 μm) with a microtome (LKB Ultratome V; LKB) and stained in 1% toluidine blue. Bright-field photographs of the cross-sections were taken using a Keyence VHX 6000 digital microscope.

For TEM, ultrathin sections (70 to 80 nm) were made with an LKB-2088 ULTROTOME V (LKB) using a diamond knife and mounted on 200-mesh copper grids. The sections were stained with 2% uranyl acetate and lead citrate and observed with a Hitachi S-4700 electron microscope with a transmitted electron detector operating at 80 kV.

## 5. Conclusions

In this study, we found that the overexpression of OsRACK1B triggers RBOH-dependent H_2_O_2_ production, leading to a systemic ROS wave in the whole plant. We also found that fertility and pollen development is severely impaired in OsRACK1B-OX plants. Based on our data, we propose that the OsRACK1B-induced ROS wave spread to the distal plant cells, leading to a change in the redox status during a highly sensitive pollen development process, ultimately affecting the fertility and seed settings in rice.

The importance of pollen development in crop yields is enormous; so, understanding the role of the RACK1-mediated pollen development pathway is very important from the scientific as well as the economic points of view. Being sessile organisms and under the global climate change scenario, plants must adapt to environmental stresses through a highly balanced and finely tuned interaction between signaling molecules. Similarly, successful reproductive growth is important for crop productivity, and various defects in reproductive organ development, such as male sterility, cause adverse crop yields. Understanding the molecular basis of ROS regulation and yield biology is the key to achieving sustainable productivity of climate-sensitive cereal crops such as rice.

## Supplementary Materials

The following supporting information [129,136] can be downloaded at: https://www.mdpi.com/article/10.3390/ijms23158455/s1.
