# Supplementary material for: Receptor for Activated C Kinase1B (OsRACK1B) Impairs Fertility in Rice through NADPH-Dependent H2O2 Signaling Pathway
Source: Int J Mol Sci. 2022 Jul 30;23(15):8455. doi: 10.3390/ijms23158455 (PMC9368841; doi:10.3390/ijms23158455)
Supplement: Supplementary file 1 [file ijms-23-08455-s001.zip › ijms-1823753-supplementary.pdf]

Supporting Information

The following Supporting Information is available for this article:

Figure S1 Identification of T-DNA insertion mutant of OsRACK1B (LOC_Os05g47890)

Figure S2 Mapping of T-DNA by Thermal Asymmetric Interlaced PCR (TAIL-PCR)

Figure S3 qRT-PCR of *OsRbohB* gene

Figure S4 BiFC analysis of the interaction between rice RACK1B and RBOHD in the Nucleus and Cytoplasm in onion epidermal cells

Figure S5 4′,6-Diamidino-2-phenylindole (DAPI)-stained pollen grains from indehiscence anthers of OsRACK1B-OX transgenic plants

Table S1 Primers used for genotyping of T-DNA insertional mutagenesis lines

Table S2 Primers used for quantitative Real Time PCR

Table S3 Primers used for TAIL-PCR

Table S4 Primers used for BiFC assay and plasmid sequencing

Methods S1 TAIL-PCR protocol

Methods S2 BiFC plasmid construction

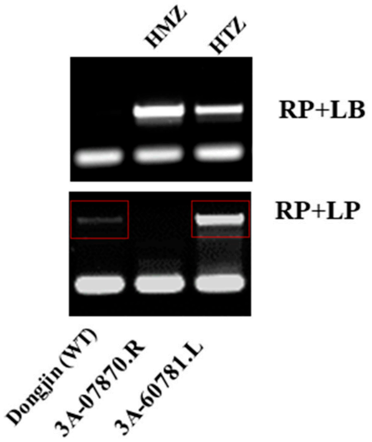

Figure S1. Identification of T-DNA insertion mutant of RACK1B (LOC_Os05g47890). Genotyping PCR of Salk lines PFG_3A-07870.R and PFG_3A-60781.L by T-DNA and gene-specific primers; Upper panel: T-DNA left border primer (LB) and a gene-specific primer (RP) amplified a ~500-bp PCR product from PFG_3A-07870.R DNA (lane 2) and PFG_3A-60871.L DNA(lane 3) but not from wild-type (WT) DNA (lane 1); Lower Panel: gene-specific primers (LP and RP) spanning the insertion site amplified a 1000-bp PCR product from wild-type DNA (lane 1, red box) and PFG_3A-60870.L DNA (lane 3, red box) but not from PFG_3A-07870.R DNA (lane 2) demonstrating homozygous (HMZ) T-DNA insertion in the PFG_3A-07870.R line and heterozygous T-DNA insertion in the PFG_3A-60871.L line.

A

B

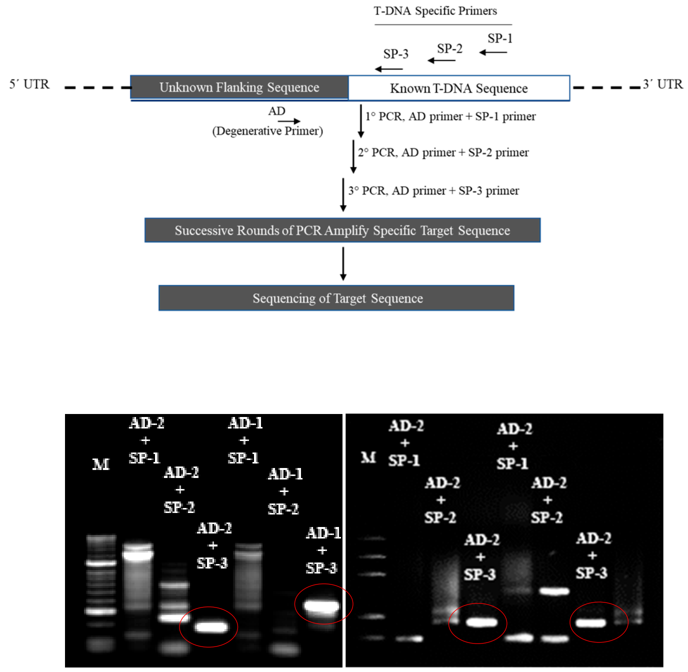

Figure S2. Mapping of T-DNA by Thermal Asymmetric Interlaced PCR (TAIL-PCR). Amplification of T-DNA flanking regions from representative OsRACK1B overexpressed rice plants using TAIL-PCR.

(A) Schematic diagram of primers design and TAIL PCR process. This strategy involves three consecutive rounds of PCR, performed with a set of three nested T-DNA specific primers (SP-1, SP-2 and SP-3) and a small, arbitrary primer, AD. The positions of three nested primers in the T-DNA region and arbitrary degenerate primer are indicated by arrows.

(B) Gel electrophoresis of Primary (1°), Secondary (2°) and Tertiary (3°) TAIL-PCR products from putative transgenic lines with two different arbitrary primers (AD-1 and AD-2) and three T-DNA specific primers. The products of the first, second and third round amplification of each transformants were shown from left to right. Lane M, DNA molecular weight marker. Circle indicates putative T-DNA insertion specific band that are excised from agarose gel for sequencing.

Methods S1

TAIL-PCR protocol: The T-DNA flanking sequence were isolated from T-DNA tagged mutants rice plants by TAIL-PCR using modified method adapted from Liu & Whittier, 1995 and Wu et al., (2015).

1° PCR reaction (μL)	2° PCR reaction (μL)	3° PCR reaction (μL)
PCR Master Mix 15<br>Dimethyl sulfoxide 1.0 (3.3%)<br>MgCl2 (50mM) 0.75<br>AD Primer(10pmol/mL) 8.0<br>SP1 Primer (10pmol/mL) 1.0<br>Genomic DNA 1.0<br>Milli-Q H_2O 3.25<br>Total 30	PCR Master Mix 15<br>DMSO 1.0 (3.3%)<br>MgCl2 (50mM) 0.75<br>AD Primer(10pmol/mL) 8.0<br>SP2 Primer (10pmol/mL) 1.0<br>1° PCR Product (diluted 100X) 1.0<br>Milli-Q H_2O 3.25<br>Total 30	PCR Master Mix 15<br>DMSO 1.0 (3.3%)<br>MgCl2 (50mM) 0.75<br>AD Primer(10pmol/mL) 8.0<br>SP3 Primer (10pmol/mL) 1.0<br>2° PCR Product (diluted 100X) 1.0<br>Milli-Q H_2O 3.25<br>Total 30
Cycle Temp. time<br><br>1 95°C 2 min<br>5 94°C 30 s<br>67°C 1 min<br>72°C 2.5 min<br>1 94°C 30 s<br>25°C 3 min;<br>ramping to 72 C, 0.3 C/s;<br>72°C 2.5 min<br>15 94°C 10 s<br>67°C 1 min<br>72°C 2.5 min<br>94°C 10 s<br>67°C 1 min<br>72°C 2.5 min<br>94°C 10 s<br>44°C 1 min<br>72°C 2.5 min<br>1 72°C 5 min	Cycle Temp. time<br><br>1 95°C 2 min<br>15 94°C 30 s<br>60°C 1 min<br>72°C 2 min<br>94°C 30 s<br>60°C 1 min<br>72°C 2 min<br>94°C 30 s<br>44°C 1 min<br>72°C 2 min<br>1 72°C 5 min	Cycle Temp. time<br><br>1 95°C 2 min<br>15 94°C 30 s<br>60°C 1 min<br>72°C 2 min<br>94°C 30 s<br>60°C 1 min<br>72°C 2 min<br>94°C 30 s<br>44°C 1 min<br>72°C 2 min<br>1 72°C 5 min<br><br>**Agarose Electrophoresis of Products:**<br>All PCR products were run in 1.5% agarose gel and desired band was gel extracted for sequencing.

Wu L, Di DW, Zhang D, Song B, Luo P, Guo GQ. 2015. Frequent problems and their resolutions by using thermal asymmetric interlaced PCR (TAIL-PCR) to clone genes in Arabidopsis T-DNA tagged mutants. *Biotechnol Biotechnol Equip* **29**(2): 260-267.

Liu Y-G, Whittier RF. 1995. Thermal asymmetric interlaced PCR: automatable amplification and sequencing of insert end fragments from P1 and YAC clones for chromosome walking. *Genomics* **25**(3): 674-681.

Methods S2: Plasmid construction for BiFC assay

Total RNA was extracted from eight-week-old wild-type rice leaves using Trizol reagent (Thermo Fisher, USA) according to the manufacturer's instructions. 1 µg of purified total RNA per sample was reverse transcribed using SuperScript™ IV VILO Master Mix kit (Thermo Fisher, USA) following manufacturers instruction. Full-length coding sequences of *OsRack1b* and 1-1300 bp of *OsRbohd* without stop codons were amplified by PCR using Q5® High-Fidelity DNA Polymerase (NEB, MA, USA). Amplicons were purified (QIAquick PCR Purification Kit, Qiagen, USA), Sanger sequenced (Genewiz, NJ) and cloned into the Gateway entry vector pCR8/GW/TOPO (Invitrogen, CA) according to the manufacturer's instruction. Orientation was confirmed by Sanger sequencing the plasmids from selected colonies. Entry clones were sub-cloned into the BiFC plasmid sets pSAT5-DEST-cEYFP(175-end)-C1(pE3130), pSAT5(A)-DEST-cEYFP(175-end)-N1 (pE3132), pSAT4(A)-DESTnEYFP(1-174)-N1 (pE3134), and pSAT4-DEST-nEYFP(1-174)-C1 (pE3136) destination vectors (https://www.bio.purdue.edu/people/faculty/gelvin/nsf/protocols_vectors.htm) using Gateway LR clonase-II enzyme mix (Invitrogen, CA). Orientation was further confirmed by Sanger sequencing of the fusion plasmids isolated (PureLink® HiPure Plasmid Midiprep Kit, Invitrogen) from selected colonies using primers listed in SI Table 4. Each pair of recombinant plasmids encoding nEYFP and cEYFP fusions was mixed 1:1 (w/w), co-bombarded with gold particles (1 um, Au, Bio-Rad) into onion epidermal layers using a Helios DNA particle delivery system (Biolistic PDS-1000/He, Bio-Rad) as described by Hollender and Liu (2010). Bombarded epidermal cells were incubated in MS liquid media with for 16–24 hr at 22°C under dark incubation, followed by observation and image analysis.

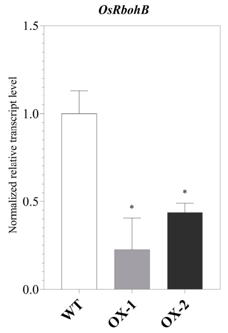

Figure S3. **The qRT-PCR analysis showing transcript level of *OsRbohB* gene.** The transcript levels were normalized using *OsActin-1*(LOC4333919). Error bars indicate the standard errors (SE) of the means of three technical repeats. Single asterisks indicate statistical significance: *P < 0.05 compared to wild-type (Student's t-test). Primers are listed in Table S2.

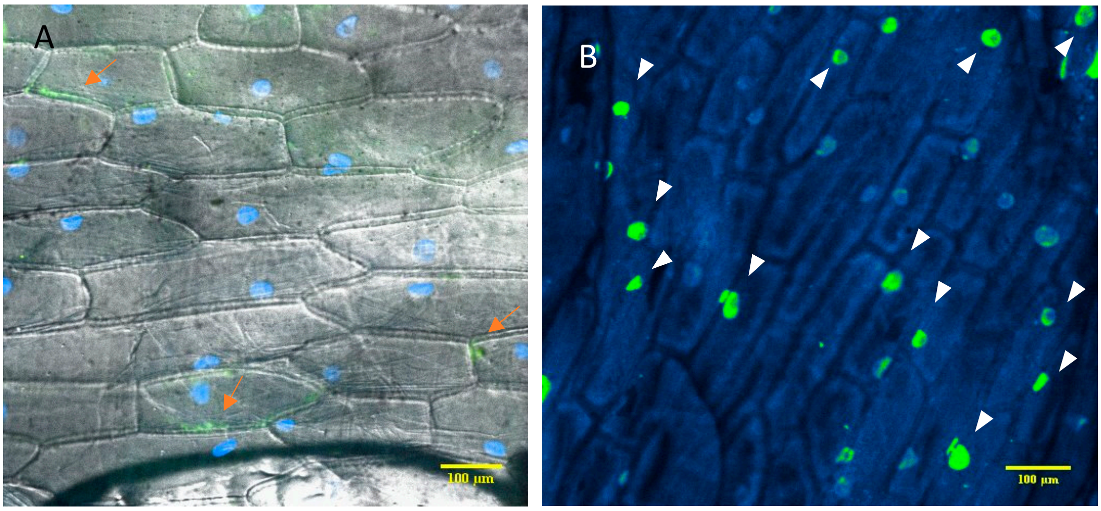

Figure S4. BiFC analysis of the interaction between rice RACK1B and RBOHD in the Nucleus and Cytoplasm in onion epidermal cells. (A) OsRACK1B and OsRBOHD interacts in the cytoplasm in normal condition. (B) The interaction complex moves to the nucleus when onion epidermal cells were incubated with salinity (200 mM NaCl) stress. Fluorescence microscopy images of onion epidermal cells co-expressing RACK1B and RBOHD fused either with the N-terminal part of YFP (nYFP) or the C-terminal part (cYFP), respectively. Images were recorded 16-24 hr after bombardment and visualized using spinning-disk confocal microscopy equipped with 10X 0.45 NA Plan Apo Lambda objective. Reconstitution of functional YFP as detected by YFP fluorescence filters (excitation 488 nm, emission 525 nm) occurs in the cytoplasm (**A**, orange arrow) in normal condition and in the nucleus (**B**, white arrowhead) during salt stress. The nuclei were stained by DAPI (4',6-diamidino-2-phenylindole, blue). Bars = 100 μm.

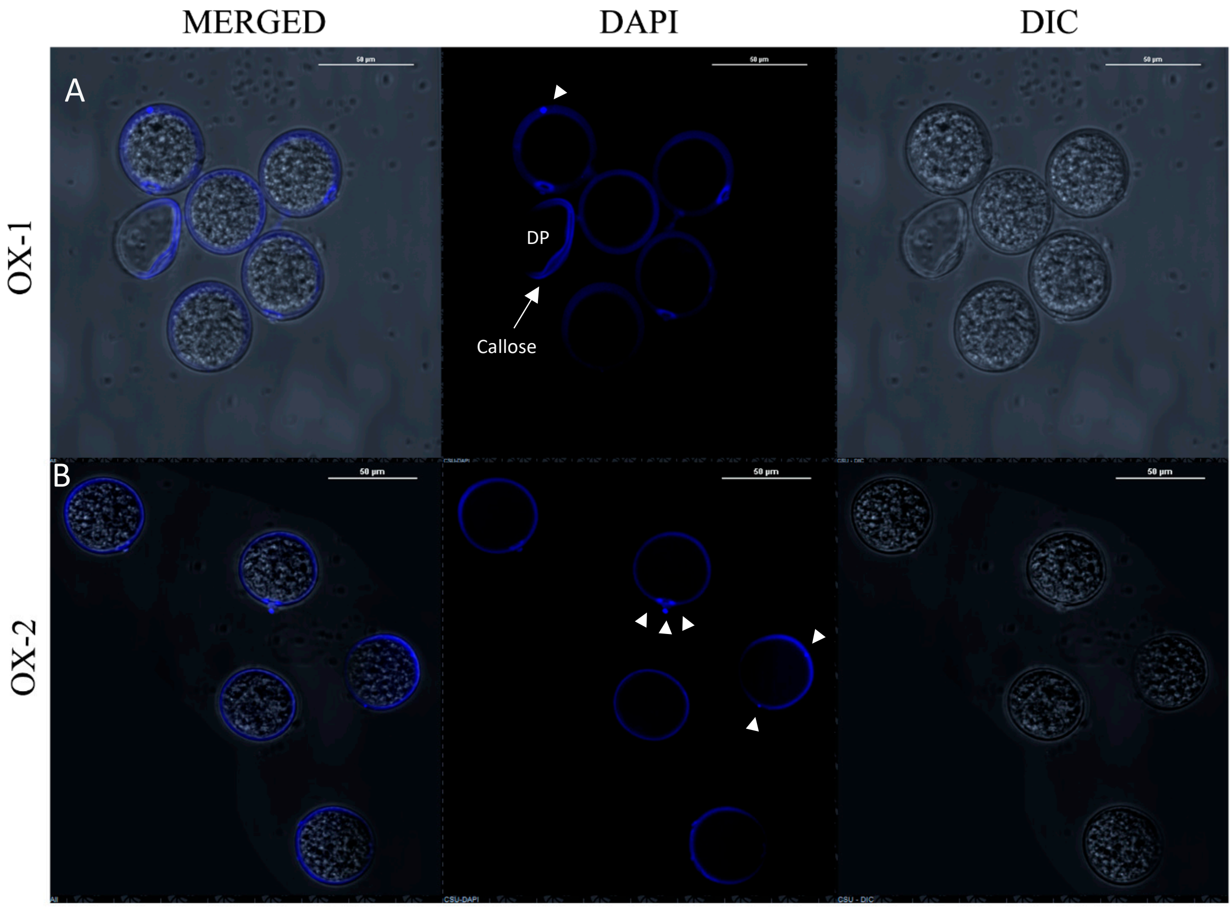

Figure S5. **4′,6-Diamidino-2-phenylindole (DAPI)-stained pollen grains from indehiscence anthers of OsRACK1B-OX transgenic plants**: (**A, top row**) Pollen from OX-1 plant showing only one single nucleate pollen (arrowhead) and a defective pollen, arrow indicates callose deposition; (**B, bottom row**) Pollen from OX-2 plant showing one normal tricellular pollen and one binucleate pollen as indicated by arrowheads. DP, Defective Pollen; Bars = 50 µm.

Table S1: Primers used for genotyping of T-DNA insertional mutagenesis lines

Primers	Insertion Chromosome	Sequence (5′→3′)	Purpose
LP	Chr05	CAGTTGAAAACAGAAGGCAGG	Genotyping of Salk line: PFG_3A-60871.L & PFG_3A-07870.R
RP		CGCACCTTCTCACTAGAGTGG	
LB+93	T-DNA left border	CTAGAGTCGAGAATTCAGTACA	

Table S2: Primers used for quantitative Real time PCR (qRT-PCR)

Gene	Accession no.	Primer Sequence (5′→3′)	Product Size (bp)
OsRACK1B	Os05g47890	Forward- ATGGCGGGCCAGGAGTCGCTCACC<br>Reverse- TCCCAGGATCCCGAGAGCGCGAACT	290
OsRbohD	Os05g38980	F-CACAAGGTTATCGCACTGACG<br>R-AGCGATGAGTATGTTGGTTGA	158
OsCatB	Os06g51150	Forward- GTTAACTACTTCCCTTCAAGGTT<br>Reverse- GCATCACACTGCGACCAGTAG	260
OsRbohB	Os01g25820	Forward- CCTAGTGGAAGAAGCTGTGCT<br>Reverse- CACTATGAAAGGGAACATCACAA	100
OsActin1	Os03g50885	Forward-TCCATCTTGGCATCTCTCAG<br>Reverse-TGGCTTAGCATTCTTGGGTC	126

Table S3: Primers used for TAIL-PCR

Primer	Sequence (5′→3′)	Tm	Purpose
AD1	NGTCGASWGANAWGAA	48	Arbitrary primer for TAIL-PCR
AD2	GTNCGASWCANAWGTT	46	
AD3	WGTGNAGWANCANAGA	44	
SP1-200	AAGTGACAGATAGCTGGGCAATG	63	Gene specific primer 200 bp from the insertion
SP2-150	GAGTAGACGAGAGTGTCGTGCTCC	64	150 bp from insertion
SP3-65	GTCCGCAATGTGTTATTAAGTTGTC	61	65 bp from insertion

Table S4: Primers used for BiFC assay and plasmid sequencing

Gene/Primer	Accession/Vector	Primer sequence (5′→3′)	Purpose
OsRbohD-1300 bp	Os05g38980	Forward- ATGGCGGGGGACTACGT<br>Rev1276-<br>GTGTCGCAAGTGTGAACGAGAA	N-terminal CDS amplification
OsRACK1B	Os05g47890	Forward- ATGGCGGGCCAGGAGTCG<br>Rev with stop-<br>CTAGATTGCATAGCCGCCAAACC<br>Rev w/o stop-<br>GATTGCATAGCCGCCAAACCCT	Full length CDS amplification
OsRACK1B-445		Forward-GGCGAGTGCAAGTACACCAT	Sanger Sequencing
		Reverse-ACCTCAAGCCAGAAGTCCAG	Sanger sequencing
GW1	TOPO TA cloning vector	GTTGCAACAAATTGATGAGCAATGC	Sequencing the insert
GW2		GTTGCAACAAATTGATGAGCAATTA	
EGFP-N	Destination Vector	CGTCGCCGTCCAGCTCGACCA	Sequencing OsRACK1B and OsRBOHD-n/c-YFP vectors
EGFP-C-FOR		CATGGTCCTGCTGGAGTTCGTG	
